# Pathophysiology, Diagnosis, and Management of Takayasu Arteritis: A Review of Current Advances

**DOI:** 10.7759/cureus.42667

**Published:** 2023-07-29

**Authors:** Sagar Bhandari, Samia Rauf R Butt, Anzal Ishfaq, Mohamed H Attaallah, Chukwuyem Ekhator, Raghu Halappa Nagaraj, Asmita Mulmi, Muhammad Kamran, Amanda Karski, Karla I Vargas, Slobodan Lazarevic, Mohammad Uzair Zaman, Gautham Lakshmipriya Vetrivendan, S M Iram Shahzed, Archana Das, Vikas Yadav, Sophia B Bellegarde, Ashraf Ullah

**Affiliations:** 1 Cardiology, Tucson Medical Center, Tucson, USA; 2 General Medicine, California Institute of Behavioral Neurosciences and Psychology, Fairfield, USA; 3 Internal Medicine, Mayo Hospital, Lahore, PAK; 4 Medicine, Cairo University, Cairo, EGY; 5 Medicine, University of Louisville, Louisville, USA; 6 Neuro-Oncology, New York Institute of Technology, College of Osteopathic Medicine, Old Westbury, USA; 7 Surgery, Avalon University School of Medicine, Willemstad, CUW; 8 Medicine, TMSS Medical College, Bogura, BGD; 9 Emergency Medicine, American University of Antigua, Miami, USA; 10 Medicine, Universidad Juárez del Estado de Durango, Durango, MEX; 11 Internal Medicine, Faculty of Medicine University of Nis, Nis , SRB; 12 Medicine, Bacha Khan Medical College, Mardan, PAK; 13 Internal Medicine, Karpaga Vinayaga Institute of Medical Sciences and Research Center, Kanchipuram, IND; 14 Internal Medicine, South Brooklyn Health, Brooklyn, USA; 15 Internal Medicine, North East Medical College and Hospital, Sylhet, BGD; 16 Internal Medicine, Pt. Bhagwat Dayal (BD) Sharma Postgraduate Institute of Medical Sciences, Rohtak, IND; 17 Pathology and Laboratory Medicine, American University of Antigua, St. John's, ATG

**Keywords:** takayasu arteritis, pathogenesis, vascular, rheumatology, review

## Abstract

Takayasu arteritis (TA) is a rare, chronic, inflammatory vasculitis that primarily affects large arteries, causing significant morbidity and mortality. This review provides an overview of the pathophysiology, diagnosis, and management of TA based on current advances in the field. TA is characterized by autoimmune-mediated inflammation, vascular remodeling, and endothelial dysfunction. The disease progresses through three stages (active, chronic, and healing phase) each presenting distinct clinical features. Diagnosis of TA can be challenging due to non-specific clinical manifestations and the lack of specific diagnostic tests. Various imaging modalities, such as angiography, ultrasound, and Doppler techniques, play a crucial role in the diagnosis of TA by visualizing arterial involvement and assessing disease extent. Management of TA involves a multidisciplinary approach, with disease-modifying anti-rheumatic drugs (DMARDs) as the cornerstone of medical therapy. Synthetic and biologic DMARDs are used to induce remission, control inflammation, and prevent complications. Non-pharmacologic interventions, such as resistance exercises and curcumin supplementation, show potential benefits. Invasive interventions, including endovascular therapy and open surgery, are used for managing vascular lesions. However, challenges remain in disease understanding and management, including the heterogeneity of disease presentation and the lack of standardized treatment guidelines. The future of TA management lies in precision medicine, utilizing biomarkers and molecular profiling to personalize treatment approaches and improve patient outcomes. Further research is needed to unravel the underlying mechanisms of TA and develop targeted therapies.

## Introduction and background

Takayasu arteritis is a rare, chronic, inflammatory vasculitis that primarily affects the large arteries, especially the aorta and its major branches. The condition was first described by Dr. Takayasu, a Japanese ophthalmologist, in 1908 [1]. Since then, significant advancements have been made in understanding the pathophysiology, diagnosis, and management of TA. This disease has a serious impact on the health and quality of life of patients. The chronic inflammation and arterial wall damage associated with the disease can lead to various complications, including stenosis, aneurysm formation, and arterial occlusion. These complications can result in organ ischemia, hypertension, and even life-threatening events such as myocardial infarction or stroke. Delayed intervention can also lead to paralysis/paresis, reduced activities of daily life, increase dependency, and affect mental health. This would emphasize the imperative need for early and advanced treatment options [2].

Vascular remodeling (segmental stenosis and/or aneurysm formation) is a hallmark feature of TA. Understanding the pathophysiology of Takayasu arteritis is crucial for developing effective diagnostic and treatment strategies. The disease is believed to have an autoimmune etiology, with immune-mediated inflammation playing a central role. An accurate and timely diagnosis of TA is essential for initiating appropriate management strategies. However, the diagnosis of TA can be challenging due to its non-specific clinical presentation and the lack of specific diagnostic tests [3]. Various imaging modalities play a crucial role in the diagnosis of TA. Angiography is considered the gold standard for visualizing arterial involvement and assessing the extent of disease [4]. It helps identify stenotic lesions, aneurysms, and vascular occlusions. Ultrasound and Doppler techniques are used for assessing arterial wall thickening, detecting flow abnormalities, and monitoring disease activity. In uncommon localizations like the vertebral arteries, the fusion of PET and MRI images may be useful in making the diagnosis of active TA.

Autoimmune-mediated inflammation, vascular remodeling, and endothelial dysfunction contribute to the pathophysiology of this disease. An accurate diagnosis relies on a combination of clinical criteria and imaging modalities. Early recognition and initiation of treatment are crucial to preventing complications and improving patient outcomes. Future research efforts should focus on unraveling the underlying mechanisms of the disease, developing novel diagnostic tools, and optimizing therapeutic strategies to improve the quality of life for individuals with TA.

## Review

Pathology and immunogenetics

**Figure 1:**
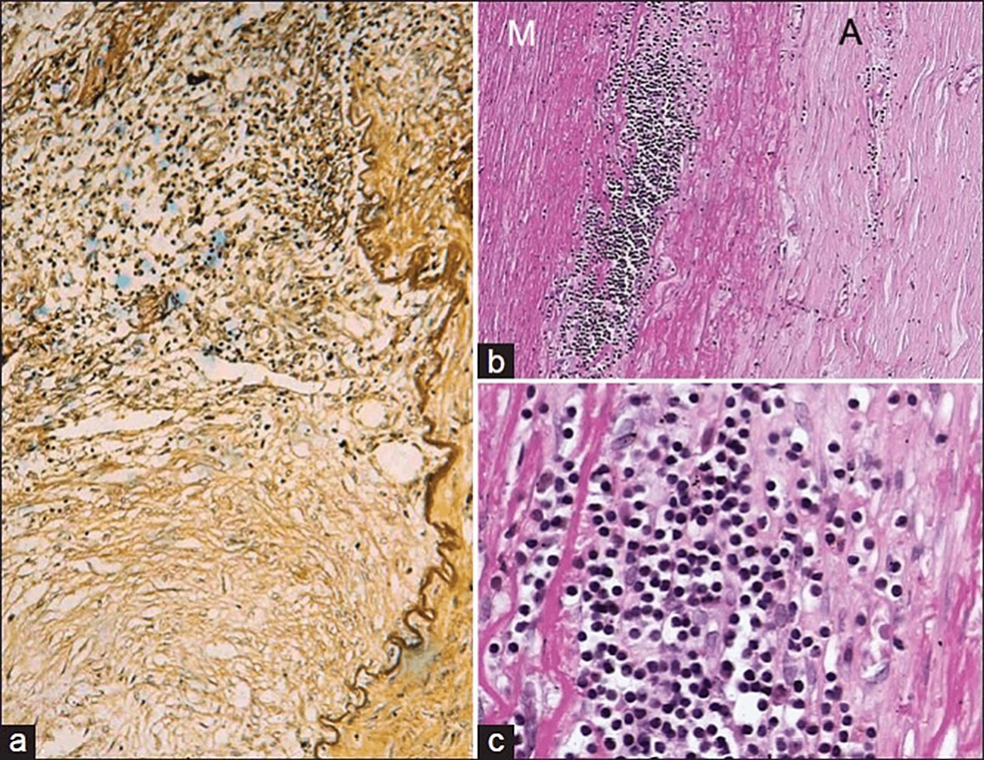
Active phase of TA in descending thoracic aorta. The images show (a) granulomatous inflammation at the medio-adventitial junction of aorta destroying the structure of an intercostal artery, (b) chronic phase of TA in descending thoracic aorta showing media (M) at the outer third with clusters of mononuclear cells. Note marked fibrosis of the adventitia (A) (hematoxylin and eosin, ×250). (c) The cells are lymphocytes, and few plasma cells and histiocytes (hematoxylin and eosin, ×250). TA: Takayasu arteritis The images are used with permission from Vaideeswar and Deshpande (2013) [5].

TA is a chronic inflammatory disease characterized by the inflammation of large arteries, particularly the aorta and its major branches. The pathophysiology of TA involves a complex interplay of immune-mediated processes, vascular remodeling, and genetic factors. TA is considered a panarteritis, with the initial site of inflammation observed around the vasa vasorum and the medio-adventitial junction [5]. In the early phase of the disease, there is active inflammation and necrosis, accompanied by mononuclear cell infiltration, including lymphocytes, histiocytes, and plasma cells, as well as edema (Figures 1a-1c). Fragmentation of elastic fibers, giant cell granulomatous reactions, and laminar medial necrosis may also be observed. These pathological features indicate the presence of an intense inflammatory response and tissue damage in the affected arteries [5,6].

**Figure 2:**
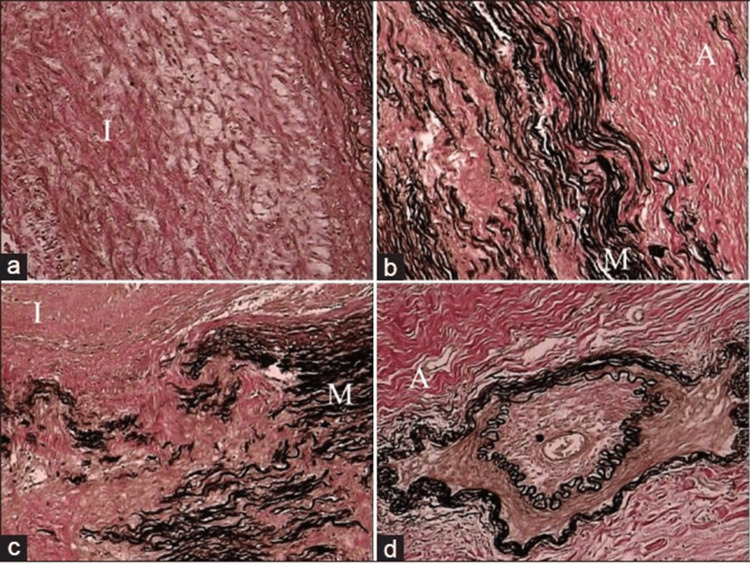
Healed phase of TA in descending thoracic aorta. The images show (a) loose fibrocellular thickening of the intima (I), (b) the junctional area between intima (I) and media (M) showing the destruction of the elastic lamellae/smooth muscle with replacement fibrosis, (c) similar features are also seen toward the outer third of the media (M) and adventitia (A), (d) fibrotic adventitia (A) showing endarteritis obliterans of a branch (elastic van Gieson, ×250). TA: Takayasu arteritis The images are used with permission from Vaideeswar and Deshpande (2013) [5].

As the disease progresses, there is reactive fibrosis and increased deposition of ground substance in the intima, along with the formation of mural thrombus and neovascularization at the intimal medial junction (Figures 1a-1c). Chronic inflammation leads to the thickening of the arterial wall and the development of stenosis and occlusion. In severe or rapid inflammation, the destruction of smooth muscle cells in the media can result in the weakening of the arterial wall, leading to vascular dilatation and aneurysm formation [5]. The healing phase of TA is characterized by adventitial fibrosis and scarring, along with persistent lymphoplasmacytic inflammation and multinucleated giant cells (Figures 2a-2d). The adventitial and periadventitial fibrosis observed in TA exceeds that seen in any other inflammatory disorder of the aorta. These structural changes in the arterial wall contribute to the long-term complications and arterial remodeling seen in TA patients [5].

The pathogenesis of TA involves a combination of genetic and immune-mediated factors. Genetic studies have identified several genetic loci associated with TA, including genes encoding human leukocyte antigen (HLA) class I and class II specificities, immune response regulators, and proinflammatory cytokines. The HLA-B52 allele has been found to be strongly associated with TA across different ethnicities [7]. Additional genetic loci within the HLA region and other non-HLA loci, such as IL12B and MLX, have also been associated with TA susceptibility [8]. Other associated genes include immune-regulatory genes (e.g., RPS9/LILRB3, LILRA3, and IL38 loci) and inflammatory cytokines (IL6 and IL12B loci) [8-10].

The immune system plays a significant role in the pathogenesis of TA, involving both adaptive and innate immune responses. Inflammatory infiltrates in TA lesions consist of macrophages and various lymphoid cells, including CD4+ and CD8+ T cells, GD T cells, natural killer (NK) cells, and B cells [11]. These infiltrates are found in close proximity to the neoangiogenic vasa vasora, which likely serves as the portal of access for immune cells to the arterial wall. The exact target of the immune response in TA remains unclear, but it is believed that vasculitogenic antigens are locally present in the arterial wall, leading to the activation and maintenance of the immune response within the arterial wall [11,12]. T helper 1 (Th1) and T helper 17 (Th17) cells responses have been implicated in both systemic and vascular manifestations of TA, although differences in specific responses exist between TA and other vasculitides like giant cell arteritis [13,14].

In conclusion, the pathophysiology of TA involves a complex interplay of immune-mediated processes, vascular remodeling, and genetic factors. The initial site of inflammation in TA is around the vasa vasorum and at the medio-adventitial junction, leading to panarteritis and subsequent arterial wall thickening, stenosis or occlusion, and aneurysm formation. Genetic factors, particularly genes encoding HLA class I and class II specificities, immune response regulators, and proinflammatory cytokines, contribute to TA susceptibility. Further research is needed to fully elucidate the underlying mechanisms and identify potential therapeutic targets for this challenging disease.

Pathogenesis

Large- and medium-sized arteries are considered immune-privileged sites that possess mechanisms to limit immune-inflammatory responses. Vascular dendritic cells (VDCs) located near the vasa vasorum act as gatekeepers, preventing the access of lymphoid cells and blunting immune responses [15]. However, in TA, activation of inherently abnormal dendritic cells breaches immune tolerance. These abnormal dendritic cells have altered the expression of regulatory receptors and overexpression of Toll-like receptors [16]. Upon exposure to unknown stimuli (environmental or viral), these dendritic cells mature and release cytokines, including IL-12, IL-23, and IL-1β. This leads to the influx of activated vasculitogenic T cells into the adventitia and media of the arterial wall [11]. The cellular infiltrate in TA releases various cytokines and growth factors, resulting in wall edema, extracellular matrix deposition, and proliferation of myofibroblasts. The activation of vasculitogenic T cells and the release of perforin and granzymes by cytotoxic CD8+ T cells contribute to acute vascular inflammation. Additionally, matrix metalloproteinases released by inflammatory macrophages contribute to granuloma formation and the disruption of the endothelial barrier [11].

Autoimmunity, particularly B cell-mediated autoimmunity, is implicated in TA. B cells also play a role in TA by promoting endothelial cell activation through the secretion of anti-endothelial cell antibodies. Anti-aorta and anti-endothelial cell antibodies (AECAs) have been detected in patients with TA. AECAs target endothelial proteins, such as the endothelial protein C receptor and scavenger receptor class B type 1, which act as negative regulators of endothelial activation. Blockade of these regulatory receptors by AECAs leads to a proinflammatory phenotype [17].

The activation of inflammatory M1 macrophages and reparative M2 macrophages is one potential mechanism underlying vascular fibrosis in TA [18]. Studies have demonstrated that during active inflammation in TA, M1 macrophages infiltrate the arterial wall, but M2 macrophages are more frequently seen during the fibrotic phase. Growth factors including platelet-derived growth factor (PDGF) and transforming growth factor beta (TGF-β) are secreted by M2 macrophages and promote wall edema, extracellular matrix deposition, and myofibroblast proliferation in the intima. However, M1 macrophages release matrix metalloproteinases and reactive oxygen species, which can weaken and degenerate the tunica media. Mast cells, known for their role in tissue repair, are also involved in the pathogenesis of vascular fibrosis in TA. Mast cell activation leads to the secretion of growth factors and cytokines, such as PDGF and TGF-β, which promote fibroblast activation and extracellular matrix deposition [11,19,20].

The mammalian target organ of rapamycin complex 1 (mTORC1) activation and Notch-1 signaling are the key mechanisms driving the activation of Th1 and Th17 lymphocytes in the inflamed arterial wall of TA [21]. Inhibition of mTORC1 activation and Notch-1 expression reduces the frequencies of Th1 and Th17 lymphocytes, potentially mitigating vascular inflammation and fibrosis [22]. Programmed cell death protein 1 (PD1) has been identified as a marker of fibrosis-driving T lymphocytes (PD1+ Th17 lymphocytes) that have been implicated in fibrosis-associated diseases and are elevated in TA [23,24]. These lymphocytes secrete TGF-β1, contributing to fibrotic processes. IL-6, a proinflammatory cytokine, plays a dual role in TA by driving vascular inflammation and fibrosis. IL-6 promotes fibroblast activation and collagen deposition through Janus kinase/signal transducers and activators of transcription (JAK-STAT) signaling pathways [25].

IL-17, produced by Th17 lymphocytes, stimulates the secretion of CYR61, a pro-fibrotic factor, in the arterial wall of TA. The interaction between IL-17 and CYR61 enhances the fibrotic phenotype [26]. Glycoprotein non-metastatic melanoma protein B (GPNMB), secreted by M2 macrophages, is associated with vascular fibrosis in TA. GPNMB promotes fibroblast activation and the expression of fibrotic markers, including collagen and matrix metalloproteinases [27].

Natural history and clinical presentation

The clinical manifestations of TA vary depending on the stage of the disease and the vascular region involved. The disease progresses through three stages: the “prepulseless” phase (stage I), the “pulseless” phase (stage II), and the “fibrotic” phase (stage III). The first two stages are common, but the triphasic pattern is only observed in 19% of patients. The clinical features include constitutional symptoms, vascular involvement (such as weak or missing pulses, vascular bruit, and vascular tenderness), and specific symptoms related to the affected vascular regions.

TA has a wide spectrum of clinical manifestations, and its diagnosis is often delayed due to the lack of specific serologic biomarkers. Depending on the disease's stage and the arteries involved, several clinical presentations of TA exist. TA is more frequently observed in Asian populations, particularly in Japan, Southeast Asia, India, and Mexico, although it has a global epidemiological distribution. With a worldwide frequency ranging from 3.2 to 40.0 cases per million and an annual incidence of 0.4 to 2.6 cases per million, the prevalence and incidence of TA fluctuate by geographic area [28,29]. There is a female preponderance in TA, with female-to-male ratios ranging from 12:1 in Turkey to 3:1 in China and India [30]. The onset of TA typically occurs between the ages of 20 and 30 years, although onset after the age of 40 years is not uncommon [29].

Patients may develop generalized symptoms in the early stages of the illness, such as fevers, myalgias, exhaustion, weight loss, night sweats, and arthralgias. These signs and symptoms are frequently linked to inflammation and might be confused with other illnesses [31]. Identifying the disease at this stage would prevent significant progression and further damage can be prevented. Vascular stenosis and narrowing increase with disease progression due to fibrosis, resulting in more specific clinical features.

In around 85% of patients, limb signs or symptoms are one of the most typical TA presentations. These include weak or missing pulses, claudication in the affected limbs, and differences in blood pressure between the right and left arms [32,33]. A total of 80-94% of patients also frequently develop vascular bruits, which are brought on by turbulence in the blood flow through stenotic arteries. These bruits frequently affect the abdominal vessels, carotid arteries, and subclavian arteries [4,34].

Hypertension is another prominent clinical feature of TA and is present in approximately 33-83% of patients [32,34]. It frequently results from renal artery stenosis, which occurs in 28-75% of patients [35]. Up to 37% of individuals develop Takayasu retinopathy, which is characterized by hypertensive alterations in the retinal blood vessels and can impair vision [30,36]. Aortic involvement is a significant complication of TA and can manifest as aortic regurgitation. It is observed in 20-24% of individuals and can be caused by dilatation of the ascending aorta, separation of the valve leaflets, and thickening of the valve. Congestive cardiac failure may occur due to hypertension, aortic regurgitation, or dilated cardiomyopathy [33].

Hypertension and/or ischemia in TA can lead to neurological symptoms. Patients may have amaurosis (visual loss), postural dizziness, and convulsions. Depending on the diagnostic technique, a fraction of individuals additionally exhibit pulmonary artery involvement, with rates varying from 14% to 100%. On chest x-rays, pulmonary vasculopathy can result in oligemic lung fields, which may assist in confirming the diagnosis of TA [36,37].

The clinical presentation of TA can vary among different populations. In Japanese patients, the disease is more common in females and is characterized by dizziness, vertigo, pulselessness, and more severe inflammation. Japanese patients also have a higher incidence of aortic regurgitation, which is due to the involvement of the aortic arch and its branches [38]. The abdominal aorta and renal arteries are affected by vasculitis in Indian patients, including a significant proportion of men, and they frequently present with headache, hypertension, and left ventricular hypertrophy [38]. It is essential to remember that TA can coexist with other illnesses. Inflammatory bowel disease and TA have been linked in studies, and individuals with TA have greater rates of spondyloarthritis and recurrent mouth ulcers [39,40]. Ocular signs are seen in 8.1-68% of patients, which varies from patient to patient. Hypertensive retinopathy and Takayasu retinopathy, caused by hypoperfusion, are the most common ocular manifestations [41].

Diagnosis

The diagnostic criteria for TA have evolved since the initial description by Ishikawa in 1988 [42]. Ishikawa proposed the first diagnostic criteria for TA, which included age <40 years at diagnosis or onset of characteristic symptoms or signs as an obligatory criterion. However, these criteria were criticized for their age restriction and low sensitivity in TA patients with predominant aortic involvement. In 1990, the American College of Rheumatology (ACR) proposed classification criteria for TA, which aimed to create homogeneous groups for research studies [43]. The ACR criteria included six parameters: age at disease onset <40 years, claudication of extremities, decreased brachial artery pulse, blood pressure difference >10 mmHg between arms, bruit over subclavian arteries or aorta, and arteriographic abnormality. A patient was considered to have TA if at least three of these six criteria were present. Although the ACR criteria were helpful for research purposes, they had limitations in clinical practice, particularly in patients with aortic involvement [44].

To address the limitations of the existing criteria, modifications were made to the Ishikawa criteria in 1995 [44]. The modified Ishikawa criteria by Sharma et al. included major and minor criteria. The major criteria consisted of specific arterial lesions, such as lesions in the subclavian arteries, common carotid artery, brachiocephalic trunk, descending thoracic aorta, abdominal aorta, and coronary arteries. The minor criteria included high erythrocyte sedimentation rate (ESR), carotid artery tenderness, hypertension, aortic regurgitation or annulo-aortic ectasia, pulmonary artery lesion, and age of coronary artery lesion before 30 years without risk factors. The presence of two major criteria or one major and two minor criteria or four minor criteria suggested a high probability of TA [44].

Imaging and angiographic classification

Imaging plays a crucial role in the diagnosis and surveillance of TA. Computed tomography angiography (CTA) is the preferred initial imaging modality due to its wide availability, better image resolution than magnetic resonance angiography (MRA), and lower cost. CTA can reveal luminal narrowing, dilatation, and changes in the vessel wall, such as wall thickening, calcification, and contrast enhancement [45]. It is particularly useful for assessing the site and extent of arterial involvement in TA.

The most widely used angiographic classification system for TA is the Numano system, proposed in 1996. The Numano classification defines six types of TA based on the topography of arterial lesions, with separate designations for coronary and pulmonary artery involvement [46]. This classification system has improved reporting standards and revealed ethnic differences in arterial lesion distribution. However, it was not formulated based on evidence and has its limitations.

Imaging modalities in diagnosis and surveillance

Various imaging modalities are utilized in the diagnosis and surveillance of TA (Table 1) [47]. Digital subtraction angiography (DSA) is considered the gold standard for diagnosis as it allows imaging of the coronary arteries and assessment of aortic pressure [48]. However, DSA is invasive and not suitable for repeated surveillance due to its associated risks.

**Table 1:** Comparative analysis of conventional imaging techniques in diagnosis and monitoring of TA. DSA: digital subtraction angiography; CTA: computed tomography angiography; MRI: magnetic resonance imaging; DUS: Doppler ultrasound; PET: positron emission tomography; TA: Takayasu arteritis

Modality	Accessibility	Ease of use	Purpose/use	Radiation	Principal limitations
DSA	Low	Low	Diagnosis/treatment	Yes	Invasive, lack of information on the vessel wall
CTA	High	High	Diagnosis/monitoring	Yes	Cannot be used in patients with renal failure or allergies to contrast media
MRI	Low	High	Diagnosis	No	Cannot be performed when some types of metals are present in the body or in patients with claustrophobia
DUS	High	High	Diagnosis/monitoring	No	Examiners’ technical proficiency strongly affects the result, subjective, acoustic shadow
PET	Low	High	Diagnosis/monitoring	Yes	Lack of criteria for positivity (FDG uptake), low resolution for small vessels

Non-invasive imaging techniques, such as 18F-fluorodeoxyglucose positron emission tomography (FDG-PET), Doppler ultrasound (DUS), computed tomography angiography (CTA), and magnetic resonance imaging (MRI), have gained importance in the diagnosis and surveillance of TA. FDG-PET, combined with CT, can localize active disease by detecting enhanced metabolic activity in the arterial wall [49,50]. However, its use in follow-up is limited due to radiation exposure and uncertain clinical significance in low-grade activity.

CTA provides detailed anatomic information and is useful for assessing arterial wall lesions. MRI, which does not involve radiation exposure, is preferred for surveillance imaging, particularly in younger patients [45]. Doppler ultrasound and contrast-enhanced ultrasound (CEUS) are also valuable tools, with Doppler ultrasound demonstrating intima-media thickness and CEUS showing neovascularization as enhancement [50,51].

Biomarkers in diagnosis and disease surveillance

Several biomarkers have been studied to aid in the diagnosis and surveillance of TA. While erythrocyte sedimentation rate (ESR) is the only biomarker included in the diagnostic criteria, other biomarkers, such as anti-endothelial cell antibodies, vascular endothelial growth factor (VEGF), IL-6, IL-8, and pentraxin 3 (PTX3), have shown promise in assessing disease activity [52-54]. However, larger cohorts and further validation are needed before these biomarkers can be routinely used in clinical practice.

Management

Management of TA involves a multidisciplinary approach and has evolved over the years with advancements in medical therapy and invasive interventions. The treatment strategies aim to control inflammation, achieve disease remission, and prevent disease-related complications. In this article, we discuss the management of Takayasu arteritis based on recent literature and the 2021 American College of Rheumatology (ACR) guidelines [55].

Medical Therapy

The use of disease-modifying anti-rheumatic drugs (DMARDs) is a cornerstone in the medical management of TA. Synthetic DMARDs, including methotrexate, azathioprine, mycophenolate, leflunomide, and cyclophosphamide, have shown efficacy in inducing remission, stabilizing vascular disease, and reducing inflammation [56,57]. Biologic DMARDs, such as tocilizumab (an anti-IL-6 receptor antibody) and tumor necrosis factor (TNF) inhibitors (infliximab, etanercept, and adalimumab), are reserved for refractory cases or when rapid control of disease activity is required. TNF inhibitors have been studied most frequently and have demonstrated at least a partial clinical response in the majority of patients [57,58]. Tocilizumab, although not meeting its primary endpoint in a randomized controlled trial, has shown promising results in reducing relapses, glucocorticoid exposure, and improving quality of life [59]. Other DMARDs, such as abatacept, ustekinumab, and rituximab, have shown varying efficacy in observational studies [57,60].

Immunosuppressive therapy is usually combined with glucocorticoids, but efforts are made to minimize steroid use due to their side effects. Lower doses of corticosteroids are recommended, and glucocorticoid monotherapy is no longer the preferred treatment strategy [61]. Combination therapy with DMARDs allows for lower cumulative glucocorticoid doses while maintaining disease control.

Non-pharmacologic Options

In addition to medical therapy, non-pharmacologic interventions (e.g., smoking cessation, healthy diet, adequate sleep, psychosocial support, counseling, therapy, etc.) can also be beneficial in managing TA. Resistance exercises have been shown to improve vascular activity scores and reduce plasma TNF-α and CRP levels in patients [62]. Curcumin, the active ingredient in turmeric, has also shown potential for improving treatment outcomes by reducing TNF-α levels [63]. However, more research is needed to establish the efficacy of these non-pharmacologic interventions.

Monitoring and Follow-Up

Frequent monitoring of clinical activity and laboratory parameters, including erythrocyte sedimentation rate (ESR) and C-reactive protein (CRP), is recommended even if a patient is in clinical remission. Treatment changes should be guided by changes in clinical status rather than asymptomatic elevations in inflammatory markers. Non-invasive imaging, such as computed tomography angiography (CTA) or magnetic resonance angiography (MRA), should be performed every three to six months early in the disease course to assess disease activity [12,64]. The frequency of imaging can be adjusted based on disease progression and response to treatment.

Invasive Therapy

Invasive therapy plays a crucial role in managing vascular lesions in TA. Endovascular therapy (ET) and open surgery are the main modalities used for invasive interventions. ET with balloon angioplasty and stenting is preferred for short lesions, while open surgery is considered for more extensive lesions or when ET is not feasible [65,66]. Invasive therapy should be performed in specialized centers with expertise in Takayasu arteritis management and a multidisciplinary team of rheumatologists, surgeons, and interventionists. It is recommended to delay invasive procedures until disease activity is controlled with immunosuppressive therapy [64,67].

Limitations in disease understanding and management

The prognosis of patients with TA has improved over the years, thanks to advancements in medical therapy and invasive interventions. The overall survival rate has increased, although patients with TA still have an increased mortality rate compared to the general population. Early diagnosis, prompt initiation of treatment, and regular follow-up are essential for better outcomes.

TA poses several challenges in terms of disease understanding and management. One significant challenge is the heterogeneity of disease presentation and outcomes. The clinical manifestations of TA can vary widely among individuals, making it difficult to establish a standardized approach to diagnosis and treatment. Some patients may present with severe vascular involvement and ischemic symptoms, while others may exhibit milder forms of the disease with less pronounced symptoms. Additionally, the disease can affect different arterial segments, leading to a wide range of complications and outcomes. This heterogeneity highlights the need for a personalized and tailored approach to patient care. A personalized approach to patient care involves a thorough evaluation of each individual's medical history, current symptoms, and disease progression. It also considers factors like age, gender, overall health, and the presence of other underlying conditions. By understanding the unique aspects of a patient's condition, healthcare professionals can develop a treatment plan that is adapted to their specific needs and challenges.

Another limitation in the management of TA is the lack of standardized treatment guidelines. Due to the rarity of the disease and the limited evidence available, there is a lack of consensus regarding the optimal treatment strategies. Current management approaches rely on immunosuppressive agents, such as corticosteroids and other immunomodulatory drugs, but the duration and dosage of treatment can vary. There is a need for more comprehensive and evidence-based guidelines to guide clinicians in the management of TA and to improve patient outcomes.

Potential for precision medicine and personalized treatment

Advancements in biomarkers and molecular profiling hold promise for the future management of TA. Biomarkers, such as cytokine levels or genetic markers, can aid in the diagnosis and monitoring of disease activity. They can also help predict treatment responses and guide treatment decisions. By identifying specific biomarkers associated with disease activity and progression, clinicians can tailor therapies to individual patients, optimizing treatment outcomes. Furthermore, molecular profiling techniques, such as gene expression profiling and genomic sequencing, can provide insights into the underlying molecular mechanisms of the disease and aid in the development of targeted therapies.

The potential for precision medicine in Takayasu arteritis extends to tailoring therapies based on individual patient characteristics. By considering factors, such as disease phenotype, genetic profile, and clinical presentation, clinicians can develop personalized treatment plans. This approach takes into account the unique aspects of each patient's disease and aims to optimize treatment response while minimizing adverse effects. The exploration of novel therapeutic targets, the role of machine learning and artificial intelligence in diagnostics, and prediction modeling can greatly contribute to the targeted therapy of TA. With the advancement of precision medicine, the management of TA can move towards a more patient-centered approach, improving overall care and outcomes for individuals with the disease.

## Conclusions

The understanding of pathophysiology, diagnosis, and management of Takayasu arteritis is of utmost importance in providing appropriate care to affected individuals. The autoimmune-mediated inflammation, vascular remodeling, and endothelial dysfunction contribute to the disease's pathophysiology. Accurate diagnosis relies on a combination of clinical criteria and imaging modalities. Early recognition and initiation of treatment are crucial to prevent complications and improve patient outcomes. Future research efforts should focus on unraveling the underlying mechanisms of the disease, developing novel diagnostic tools, and optimizing therapeutic strategies to improve the quality of life for individuals with Takayasu arteritis.
